# Alcohol Use and Its Associations With Frailty, Fractures, and Falls Among Older Adults With HIV

**DOI:** 10.35946/arcr.v45.1.08

**Published:** 2025-08-08

**Authors:** Derek D. Satre, Verena E. Metz, Natalia Van Doren, Michael J. Silverberg, Jennifer O. Lam

**Affiliations:** 1 Department of Psychiatry and Behavioral Sciences, Weill Institute for Neurosciences, University of California, San Francisco, California; 2 Division of Research, Kaiser Permanente Northern California, Pleasanton, California; 3 Department of Health Systems Science, Kaiser Permanente Bernard J. Tyson School of Medicine, Pasadena, California

**Keywords:** alcohol, HIV, aging, older adults, frailty, fractures, falls

## Abstract

**PURPOSE:**

More than half of people with HIV (PWH) in the United States are now over age 50. Although alcohol consumption declines with age among PWH, as it does in the general population, alcohol misuse and alcohol use disorder (AUD) pose substantial health risks. Aging leads to increased sensitivity to alcohol due to slower metabolism, central nervous system changes, less lean body mass, greater prevalence of co-occurring medical conditions, and polypharmacy (simultaneous use of multiple medicines). These vulnerabilities heighten the adverse effects of alcohol use among older PWH compared with both younger PWH and people without HIV of all ages. This review examines associations between alcohol use and three interrelated health outcomes of growing research interest—frailty, fractures, and falls—each of which has substantial negative impacts on longevity and quality of life among PWH and may be influenced by alcohol use.

**SEARCH METHODS:**

Search terms included alcohol, drinking, binge drinking, heavy drinking, AUD, alcohol abuse, alcohol dependence, problematic alcohol use, mild/moderate alcohol use, high-intensity drinking, risky drinking, alcoholism, frailty, falls, fractures, HIV, PWH, PLWH, ART, and AIDS. All studies included PWH age 50 and over. In June 2024, authors identified original studies published in English between June 1, 2014, and June 1, 2024, by searching PubMed, Web of Science, and ScienceDirect and reviewing reference lists of reviews and meta-analyses identified in the search.

**SEARCH RESULTS:**

Searches yielded a total of 512 articles; 114 duplicates were removed. Two independent reviewers screened the abstracts of the remaining 398 articles, of which 326 articles were excluded based on having inappropriate sampling, exposures, or outcome measures. Seventy-two articles underwent full-text review; of these, 14 articles met inclusion criteria and 58 articles were excluded. Reasons for exclusion were wrong outcomes (*n* = 12), no drinking or alcohol quantification (*n* = 15), wrong population (*n* = 8), outside of timeframe (*n* = 3), not in English (*n* = 2), wrong frailty measure (*n* = 9), and review/meta-analysis (*n* = 9).

**DISCUSSION AND CONCLUSIONS:**

Results across the 14 studies indicated that, among older PWH, greater alcohol use was linked to higher risk of frailty, fractures, and falls. However, evidence was limited, with most literature based on observational studies. Important future potential research directions include longitudinal assessments of alcohol–frailty relationships among PWH age 50 and over; evaluating the role of alcohol use in the development and progression of frailty accounting for mid- and later-life comorbidity and resultant polypharmacy; and examining moderators of the alcohol–frailty relationship. Future research examining interventions to reduce risk of frailty, fractures, and falls among aging PWH also would be beneficial.

Effective antiretroviral therapy (ART) has improved the longevity of people with HIV (PWH).^1^ In the United States, most PWH are over age 50 and life expectancy is nearing that of people without HIV (PWoH).^2^ As people with HIV (PWH) get older, they face unique health challenges. Compared with PWoH, older PWH are more likely to experience chronic physical and mental health conditions, including cancer, cardiovascular disease, dementia, and depression, which are often exacerbated by alcohol use.^3^, ^4^ While elevated risk of comorbidity among older PWH is well-documented, less is understood about the combined impact of alcohol use and HIV infection on frailty, fractures, and falls,^5^–^7^ serious health outcomes often associated with mortality.

Alcohol also adversely impacts the health of older PWH through several biological and behavioral pathways. Animal models have shown that chronic alcohol use induces oxidative stress and mitochondrial dysfunction, leading to cellular damage.^8^ Alcohol contributes to immune senescence via inflammation driven by gut permeability and microbial translocation.^7^ Studies using simian immunodeficiency virus (SIV)-infected macaque models have shown that chronic alcohol exposure amplified viral replication and immune activation, even when viral load was controlled.^9^ In humans, drinking alcohol poses specific risks to older PWH, as HIV exacerbates alcohol’s damaging effects on the liver, increases inflammation, and worsens nutritional deficiencies.^10^, ^11^ Alcohol use also leads to worse retention in care, lower rates of viral suppression,^3^,^12^,^13^ and higher HIV disease severity.^14^ Moreover, older PWH are frequently prescribed multiple medications (i.e., polypharmacy), many of which have adverse interactions with alcohol.^15^

Alcohol misuse spans a continuum, from exceeding National Institute on Alcohol Abuse and Alcoholism (NIAAA)-recommended limits for daily and/or weekly number of drinks to alcohol use disorder (AUD).^16^ Alcohol misuse across this spectrum is common in PWH, occurring in up to a quarter of this population.^17^, ^18^ PWH also may experience harms at lower levels of consumption compared to PWoH.^19^

The relationship of alcohol use to frailty, fractures, and falls among older PWH is a relatively new area of public health interest that merits further attention given the rapidly growing population of older PWH. Frailty is itself a relatively new research area and lacks a consensus definition.^20^ It has been conceptualized in multiple ways, including as accumulation of deficits,^21^ decrease in available energy,^22^ or a state of vulnerability to stressor events resulting from declines in multiple physiological systems.^6^, ^23^ Compared with the general population, PWH are at higher risk for frailty and are more likely to become frail at younger ages.^24^, ^25^ Frailty among PWH previously has been associated with severe immunodeficiency and AIDS. In the era of ART, frailty has increasingly been linked to age-related comorbidities, including cardiovascular disease, kidney disease, diabetes, osteoporosis, cognitive impairment, and cancers,^26^ as well as smoking, injection drug use, and other substance use.^11^,^27^–^30^ Consequences of frailty include recurrent falls, fractures, hospitalization, and increased mortality risk.^31^–^33^ As the population of PWH ages, understanding and addressing modifiable risk factors for frailty (and its precursor, prefrailty) are critical for improving overall health and quality of life.^34^

Frailty among PWH has been assessed using several tools. Among the most common, the Fried frailty phenotype includes five criteria: weight loss, weakness or poor handgrip strength, exhaustion, slow walking speed, and low physical activity.^35^ The Rockwood Frailty Index is based on a proportion of accumulated deficits, including symptoms, signs, functional impairments, and laboratory abnormalities.^36^ The 58-item Deficit Index (DI58) also is based on the model of accumulated deficits, in which AIDS-defining deficits have been removed to enable generalizability between PWH and PWoH.^37^ Finally, the Veterans Aging Cohort Study (VACS) Index 1.0 is a score calculated based on routine clinical laboratory tests combined with patient age. To improve its ability to discriminate changing individual mortality risk, the VACS Index 2.0 includes added clinical predictors (body mass index, total white blood cell count, and albumin) and the use of continuous instead of categorical variables.^38^ Originally developed for predicting mortality among PWH,^39^ the VACS indices can also be meaningful measures of frailty^19^, ^33^ and are predictive of fractures and falls.^40^

Fractures have been considered in the literature variously as indicative of frailty or as potential consequences.^32^ PWH experience significantly more fractures than the general population^33^, ^41^ and these occur at younger ages.^42^ Known risk factors include body mass index, smoking, drug use, polypharmacy, frailty, and hepatitis C/HIV coinfection.^43^ Alcohol use may also contribute but remains understudied.^44^, ^45^

Fall risk increases with frailty and often results in fractures. Falls also have been directly associated with alcohol use frequency and quantity among older PWoH.^46^–^48^ Alcohol impairs balance, concentration, and spatial awareness and increases risk of delirium.^49^ Because PWH are more likely to have osteoporosis than PWoH,^50^ especially postmenopausal women,^51^ fall risk reduction is even more critical for preventing loss of function in this population.^52^ Recurrent falls are more likely among PWH who are frail or who have poor balance, gait, and endurance,^53^ and alcohol use may also pose a risk. A useful conceptual model that integrates the potential relationship of alcohol to frailty and falls among older PWH and includes associated biological mechanisms, has been proposed by Womack and Justice.^54^ Building on this work, a proposed model that identifies additional considerations (i.e., behavioral factors, comorbidities, and medication concerns) may aid in understanding the role of alcohol in leading to these adverse outcomes (see Figure 1).

Given the increasing number of older PWH and the risks associated with alcohol use in this population, there is a critical need to synthesize current research on how alcohol impacts frailty, fractures, and falls. The objective of this review was to compile and analyze the existing literature assessing these specific consequences, to synthesize current research and identify knowledge gaps to inform future research priorities.

## Methods

### Search Strategy

In June 2024, a systematic literature search was conducted in PubMed, Web of Science, and ScienceDirect. Search terms included alcohol, drinking, binge drinking, heavy drinking, AUD, alcohol abuse, alcohol dependence, problematic alcohol use, mild/moderate alcohol use, high-intensity drinking, risky drinking, alcoholism; frailty, falls, fracture; older adults/patients/subjects/participants, geriatric; HIV-positive, HIV+, PWH, AIDS, HIV infection, ART, PLWH. Authors identified additional relevant papers by reviewing the reference lists of reviews and meta-analyses identified using these search terms. Searches were saved in EndNote (version 21) and uploaded into Covidence systematic review managing software.^55^

To be included in the review, studies needed to include PWH age 50 and over, with a mean age of 45 or older to ensure that the studies included older PWH while accommodating research that included participants slightly younger than age 50. Papers needed to have been published in English (due to lack of translation resources), and between June 1, 2014, and June 1, 2024, because this time period represents the “treat all” era, when the World Health Organization recommended treating all PWH regardless of CD4+ T cell count.^56^ Two phases of assessment were conducted: The initial abstract screening excluded records with clearly incorrect sampling, exposure, or outcomes. This was followed by full-text review, which excluded papers with wrong outcomes, no drinking or alcohol quantification, wrong populations, or wrong frailty measures and papers that were published outside of the timeframe, were not in English, or were reviews or meta-analyses (see Figure 2).

### Bias Assessment

The Risk Of Bias In Non-randomized Studies–of Exposure (ROBINS-E)^57^ tool was used to assess the selected articles. ROBINS-E provides a structured approach to assessing bias in observational studies. It includes seven domains of bias, each of which is addressed using a series of signaling questions that aim to gather information about the study and the analysis being assessed. Domains include confounding, exposure measurement, participant selection, interventions, missing data, outcome measurement, and selection of reported results. Overall risk-of-bias judgment is based on the domain with the greatest risk of bias. After completing the relevant signaling questions, two authors made judgments for each included study, and overall risk of bias was categorized as low, unclear, or high. Final ratings are reported in Appendix 1.

## Results

### Literature Search and Selection of Papers for Inclusion

Database and citation searches yielded 512 published studies, with 114 duplicates removed before screening. Two independent reviewers screened the abstracts of 398 references to identify articles that met search criteria, after which 326 were excluded (see Figure 2). Seventy-two records underwent full-text review, of which 58 papers were excluded and 14 were included for data extraction. Reasons for exclusion at the full-text review stage are listed in Figure 2. Both review stages were conducted by two independent reviewers; in case of conflict, a third reviewer resolved the conflict.

### Description of Studies

The review identified 14 studies published between 2014 and 2024 in samples that included older PWH (age 50 and over), conducted primarily in the United States (see Appendix 1). Seven studies assessed frailty outcomes, five had fracture outcomes, and four had fall outcomes; two studies reported on both fractures and falls. Alcohol use measures included quantity/frequency questions, original and shortened Alcohol Use Disorders Identification Test (AUDIT and AUDIT-C), and AUD diagnoses. Frailty assessments included the VACS Index,^19^ DI58,^37^ and Fried frailty phenotype.^35^ Fracture and fall outcomes included self-report measures,^58^ electronic health record (EHR)-based diagnoses, and radiology reports.^59^

### Association of Alcohol Use and Frailty

Three analyses from the New Orleans Alcohol Use in HIV (NOAH) study of PWH engaged in care examined relationships between lifetime and recent alcohol use and frailty outcomes.^37^,^60^,^61^ In the first study, Maffei et al.^37^ calculated alcohol exposure using the Lifetime Drinking History instrument,^62^ and recent (30-day) exposure using timeline follow back (TLFB).^63^ Results showed that after adjustment, lifetime alcohol exposure was associated with DI58 and Fried frailty phenotype, but not with VACS Index 2.0 score.

A second NOAH analysis examined patterns of prior alcohol use in relation to subsequent frailty outcomes.^60^ Results showed that a steeper increase in alcohol consumption after ages 10 to 20 through midlife (age 40) was associated with greater frailty on the DI58, lower health-related quality of life, and greater morbidity. This may indicate that the rate of increase in alcohol use earlier in life has the potential to impact subsequent frailty.

A third examination of NOAH study data focused on the relationship of body composition to frailty, with alcohol as a potential moderator.^61^ Recent alcohol use, measured by phosphatidylethanol (PEth) levels, moderated the relationship between lean body mass (assessed with the fat-free mass index) and frailty as indicated by DI58 score. Regarding moderation effects, the authors hypothesized that exercise-based interventions to preserve muscle mass could protect against frailty.

A study by the Centers for AIDS Research Network of Integrated Clinical Systems collaboration^64^ included both current and retrospective measures of alcohol use and AUD. The outcome was a modified Fried frailty phenotype.^65^ Results showed that in bivariate comparisons, frailty was more prevalent among PWH who no longer drank alcohol but had a history of AUD (18%) compared with other categories of alcohol use (11% to 16%). However, in multivariate analyses, both current nonhazardous alcohol use (defined as AUDIT-C scores of 1 to 4 for men, 1 to 3 for women) and hazardous alcohol use (AUDIT-C score of ≥ 5 for men and ≥ 4 for women) were associated with lower risks of being prefrail and frail. The authors noted that this pattern of findings, as in the study by Maffei et al.,^37^ was consistent with the hypothesis that people with heavy drinking or AUD earlier in life experienced longer-term risks to health even if they stopped drinking, and that cross-sectional examination of associations between alcohol use and frailty could lead to incorrect inferences that alcohol use is protective.

In an analysis of VACS data on male study enrollees, alcohol exposure was assessed with AUDIT-C during primary care screening, and frailty measurement was based on the VACS Index 1.0 using laboratory values closest to the AUDIT-C date (± 90 days).^19^ Results showed that VACS Index 1.0 scores increased with higher AUDIT-C scores, number of drinks per month, and greater frequency of heavy episodic drinking (defined as six or more drinks per occasion). In linear regression models, PWH with AUDIT-C scores of 5 to 7 had significantly higher VACS Index 1.0 scores than people with AUDIT-C scores of 1 to 4. Analyses also showed that PWH experienced frailty at lower levels of alcohol use compared with PWoH, and the authors concluded that alcohol consumption limits should be lower among PWH.

A cross-sectional study of treatment-receiving PWH in an urban HIV care setting in Canada extracted 29 clinical indicators from the EHR to compute a frailty index.^66^ Latent class analyses identified four index subtypes: Subtype 1 (severe metabolic dysfunction + polypharmacy), Subtype 2 (less severe metabolic dysfunction + polypharmacy), Subtype 3 (lung and liver dysfunction + polypharmacy) and Subtype 4 (least severe metabolic dysfunction). Membership in Subtype 3 was associated with heavy alcohol use (defined as more than nine drinks per week for women and more than 14 drinks per week for men), smoking, and using crack/cocaine. Compared with abstinence, nonheavy alcohol use was associated with lower risk of frailty.

Lastly, a cross-sectional study of 415 PWH with a history of injection drug use that used the Fried frailty phenotype found a trend for hazardous alcohol use (AUDIT ≥ 8) to be higher among frail (25%) and prefrail (24%) PWH compared with robust (19%) PWH, although significance was not reported.^67^

### Association of Alcohol Use With Fractures and Falls

The Boston Alcohol Research Collaboration on HIV/AIDS (ARCH) Frailty, Functional Impairment, Falls, and Fractures (4F) Study examined prospective relations between alcohol consumption, fractures, and bone mineral density over a 3.5-year time period in PWH with substance use disorder or a history of injection drug use.^58^ In adjusted models, no associations were found between annual alcohol consumption and incident fractures. Alcohol use was assessed as mean grams per day of alcohol, mean number of heavy drinking days (five or more drinks for males and four or more drinks for females) per month, and any heavy drinking, measured using the 30-day TLFB twice per year.

Another Boston ARCH 4F Study examined the relationship between alcohol use and fracture and fall risk in a sample of PWH (*N* = 251).^68^ Alcohol use measures included any past 14-day heavy use, average amount of alcohol use per day, and days of heavy use (five or more drinks for males and four or more drinks for females). The primary outcome was reporting any falls. Secondary outcomes were number of falls (one, two, three, or more), fracture (any), and any fall- or fracture-related emergency department visit or hospitalization. Based on adjusted analyses, heavy alcohol use was associated with self-reports of having a fall, multiple falls, and fall- or fracture-related emergency department visit or hospitalization. Higher average amount of alcohol consumed per day and more days of heavy use were associated with multiple falls.

Another study that included Boston ARCH participants as well as PWH in St. Petersburg, Russia, found that 35% (87 of 251) of PWH in Boston (mean age 52 years; standard deviation [SD] = 10) and 12% (46 of 400) of PWH in St. Petersburg (mean age 39 years; SD = 6) reported a fall.^69^ Cohort members reported alcohol use problems of a range of levels, as well as other substance use. Most of the participants in the Russian cohort met criteria for a moderate or severe AUD (64%, 256 of 400 participants), whereas only a minority did in the Boston cohort (27%, 66 of 251 participants). Analysis of the relationship of AUD severity to fall risk found that each additional AUD criterion was significantly associated with a fall in both cohorts. Compared with no heavy alcohol use (more than six drinks/occasion), any heavy alcohol use was associated with more than twice the odds of a fall in Boston. These analyses adjusted for other substance use, depression, and functional status.

Three studies have examined alcohol use, fractures, and falls in the VACS. In a nested, case-control study of PWH and a matched cohort of PWoH, Womack et al. found that hazardous alcohol use (defined by AUDIT-C score) was associated with serious falls (defined based on a combination of *International Classification of Diseases, Ninth Edition* [ICD-9] diagnoses and radiology reports), with no difference by HIV status.^59^ A subsequent longitudinal study examined associations between AUD diagnosis and serious falls (i.e., falls significant enough to result in a visit to a health care provider) and subsequent fragility fractures (i.e., hip, vertebral, and upper arm fractures) using ICD-9 codes and radiology reports.^70^ AUD was more prevalent among individuals who ever fell (25%) compared to those who never fell (19%). Moreover, AUD increased the odds of having fragility fractures after a fall.

A third VACS-based study analyzed modifiable risk factors contributing to serious falls and fragility fractures among U.S. veterans (*N* = 21,041) from 2010 to 2015.^40^ AUD diagnosis and hazardous alcohol use (defined based on AUDIT-C score) increased the risk of a serious fall by 4% and 3%, respectively. Results for fragility fractures were even more striking: AUD was the second-highest contributor to risk for fragility fracture at 8%, second only to opioid prescriptions. Hazardous alcohol use had a similar contribution (3%) to fragility fractures as to serious falls. Finally, substance use (elevated AUDIT-C score, or diagnosis of AUD or substance use disorder) accounted for 16% of fragility fracture risk.

In a study using the French Hospital Database on HIV, Costagliola examined the effect of ART on osteoporotic fractures, with alcohol consumption also assessed.^71^ Among the 254 participants with reported fractures, 200 (79%) had had only one fracture, 37 (15%) had had two fractures, 11 (4%) had had three fractures, and six (2%) had had four fractures. There were 69 fractures in the hip, 53 in the spine, 51 in the wrist, 30 in the humerus upper end, 14 in the tibia upper end, 11 in the femur lower end, six simultaneous fractures of three ribs, and 20 other fractures. In a bivariate comparison of PWH with fractures and case controls without fractures who were matched for age, sex, and time period of HIV diagnosis, the study found that alcohol consumption of more than two glasses/day was significantly higher in PWH with fracture than in PWH without fracture.

### Assessment of Risk of Bias

Risk of bias across the identified studies was variable, as included studies applied observational designs, but bias levels primarily were in the low to moderate range (see Appendix 1). Risk of bias was considered moderate or high when alcohol was only one of the exposures measured or was imputed over longer periods of time (e.g., lifetime) based on self-report, if appropriate covariates were not included, or when alcohol use was not assessed using validated measures.

## Discussion

This narrative review represents the first known attempt to examine the literature on relationships between alcohol use and frailty, fractures, and falls among older PWH. Findings across 14 studies suggest that greater alcohol use is associated with higher risk of frailty, fractures, and falls. Although this review provides the foundation for more research on these often-overlooked risks, the evidence base remains limited—particularly in isolating independent associations after adjusting for confounding variables, such as demographic characteristics, smoking, and other substance use. Despite this, valuable insights can be gleaned from the current review, as detailed below.

Overall, findings suggest that higher lifetime alcohol use is positively associated with frailty outcomes as measured by frailty index scores and the Fried frailty phenotype. For example, Maffei et al.^37^ and Madkour et al.^60^ found that higher lifetime alcohol use was positively associated with frailty, indicating that cumulative alcohol exposure may play a significant role in frailty development. Additionally, Crane et al. reported that frailty was more prevalent among PWH who had a history of AUD but no longer drank, compared with those who were current drinkers.^64^ This may support the frequently observed J-shaped curve pattern, while perhaps emphasizing that early and lifetime alcohol exposure could be more impactful than current drinking in terms of frailty risk. Yet, continued consumption may also be harmful—for example, due to inflammation resulting from microbial translocation.^7^

Review results indicate that the relationship between alcohol use and fractures or falls has received less attention compared with frailty. Womack et al. found that AUD significantly increased the odds of fragility fractures following serious falls.^70^ This was echoed by Womack et al., who reported that AUD was linked to both osteoporotic and hip fractures.^40^ Kim et al. also noted that heavy alcohol use was associated with a higher likelihood of falls, multiple falls, and fall-related emergency department visits, further underscoring the role of alcohol as a modifiable risk factor for these outcomes.^68^ However, not all studies identified a direct link between alcohol consumption and fractures. For example, Saitz et al. reported no significant association between alcohol consumption and changes in bone mineral density or fractures.^58^ This variability highlights the potential benefits of longitudinal studies that can better capture the cumulative impact of alcohol use on bone health and fracture risk in this population.

### Sex and Demographic Differences

One notable limitation of the studies included in this review is the lack of sex diversity in the samples, as most of the research has focused on male PWH. Although PWH in the United States are predominantly male, it is important to recognize that alcohol use may affect female PWH differently. For example, research suggests that females may be more vulnerable to the adverse health effects of alcohol,^72^, ^73^ which could plausibly translate to faster progression to frailty and associated consequences (i.e., fractures and falls) in female PWH. Postmenopausal bone changes are an important contributing factor in sex differences in aging.^74^ Future research should aim to include more female participants to provide a more comprehensive understanding of alcohol’s effects in PWH specifically, in addition to examining frailty and fall risk by HIV status among females.^75^–^77^ A strength of the literature is the inclusion of substantial numbers of non-White, especially Black PWH, due to the focus on effects of alcohol, HIV, and aging in the NOAH, Boston ARCH, and VACS samples. Future work may consider greater efforts to include larger numbers of other racial/ethnic groups, especially older adult Latino PWH, who often have worse HIV care and health outcomes in the United States.^78^, ^79^

### Data Quality, Risk of Bias, and Measurement Issues

The quality of the studies included in this review varied, with half of the studies categorized as having a moderate to high risk of bias based on a standard evaluation tool for nonrandomized studies (i.e., the ROBINS-E tool). The high bias-risk assessments were mainly driven by bias due to potential confounding; in many studies, it was not possible to control for all confounding factors and/or to even assess all confounding factors, especially when (alcohol) exposures happened over long periods of time. Despite rigorous statistical analyses, confounding bias often remained.

Heterogeneity in frailty definitions complicates the ability to draw generalized conclusions about the direct effects of alcohol on this condition, reflecting a broader challenge within aging research that impacts both the interpretation and comparability of results across different studies.^23^,^80^,^81^ Recent reviews have emphasized that despite numerous operational definitions, a unified approach to frailty is yet to be achieved.^82^ Despite this variability in definitions, this review utilized standard measures, such as the VACS Index, DI58, and the Fried frailty phenotype that have been extensively validated. Yet frailty was generally measured at single time points and often as a binary outcome. Because frailty is dynamic and potentially modifiable, important future directions include examining frailty trajectories, transitions, and/or severity.

An additional concern is the inconsistency in how alcohol use was measured and categorized across studies. Many studies relied on broad categories, such AUD diagnoses, although many also had more detailed measures of alcohol use (e.g., quantity and frequency). Few studies used PEth in addition to self-report measures.^37^, ^61^ Although PEth has a brief (approximately 2-week) time window for alcohol use detection,^83^ it is useful for validating self-report and for studies that aim to assess the potential impact of recent drinking. Given the variability in alcohol measurement, future research could be improved by prioritizing consistent assessments, such as the AUDIT-C and PEth, to enhance the comparability. Nevertheless, the overall consistency of results across studies despite the variability in measurements strengthens the findings of strong associations between alcohol use and clinical outcomes in this population.

The “sick quitter” artifact observed in prior literature— which suggests that an apparent positive relationship between alcohol use and health outcomes stems from the fact that people in worse health often decrease drinking^84^—also poses a challenge in assessing the relationship of alcohol use with frailty and related outcomes.^85^ However, a growing number of studies, including studies noted in this review, have helped to clarify the impact of alcohol use on adverse health effects by including measures of alcohol use earlier in time (e.g., retrospective assessment of cumulative alcohol use) in combination with subsequent frailty indicators.^60^

### Gaps in the Literature and Future Directions for Research

While the relationship between alcohol use and frailty has been explored in cross-sectional and retrospective studies, longitudinal studies are lacking. Better understanding whether decreases in alcohol use frequency and/or intensity prevent, delay, or reverse frailty could inform intervention approaches. The impact of alcohol use reduction on risk of fractures and falls among PWH has also not been studied longitudinally. Older age could potentially modify the association between alcohol use and frailty, fracture, and fall outcomes in PWH. This represents a significant gap in the current literature and underscores the need for longitudinal studies that can address potential age-related effects.

The role of neurological impairments in mediating or moderating the effects of alcohol use on frailty, falls, and fractures also has rarely been explored. Given that older PWH are more likely to experience neurocognitive impairments and age-associated dementias than age-matched peers,^86^, ^87^ evaluating how alcohol use may jointly impact trajectories of cognitive and physical functioning could shed light on processes underlying vulnerability to frailty among older PWH.^88^ While alcohol use has significant implications for aging, other factors that reduce cognitive functioning also can increase risk of falls and fractures. Improved knowledge about potential interrelated pathways could inform integrated prevention efforts, such as interventions that combine physical and cognitive training.^89^ Yet, assessment of the relative contributions of these factors to risks of frailty, falls, and fractures, particularly in the context of alcohol use, is still a relatively new area of research.^90^ Other related factors observed in PWH include peripheral neuropathies,^91^ sarcopenia,^92^ and aging-related changes in function that affect strength, balance, gait, stance, or postural control. These conditions increase in prevalence with age and may be exacerbated by alcohol use. Acute intoxication and alcohol use intensity likely contribute to fall, fracture, and other injury risk,^93^, ^94^ but this review did not identify any studies that examined these associations among older PWH.

**Figure 1:**
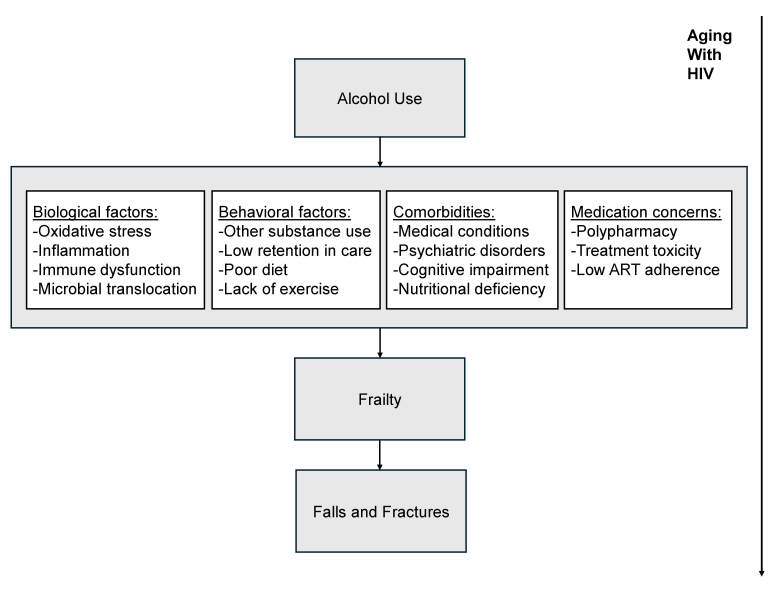
Conceptual model of how alcohol use may contribute to frailty, fracture, and fall outcomes among older adults with HIV. *Source:* Adapted from Womack JA, et al., 2020.^54^

**Figure 2:**
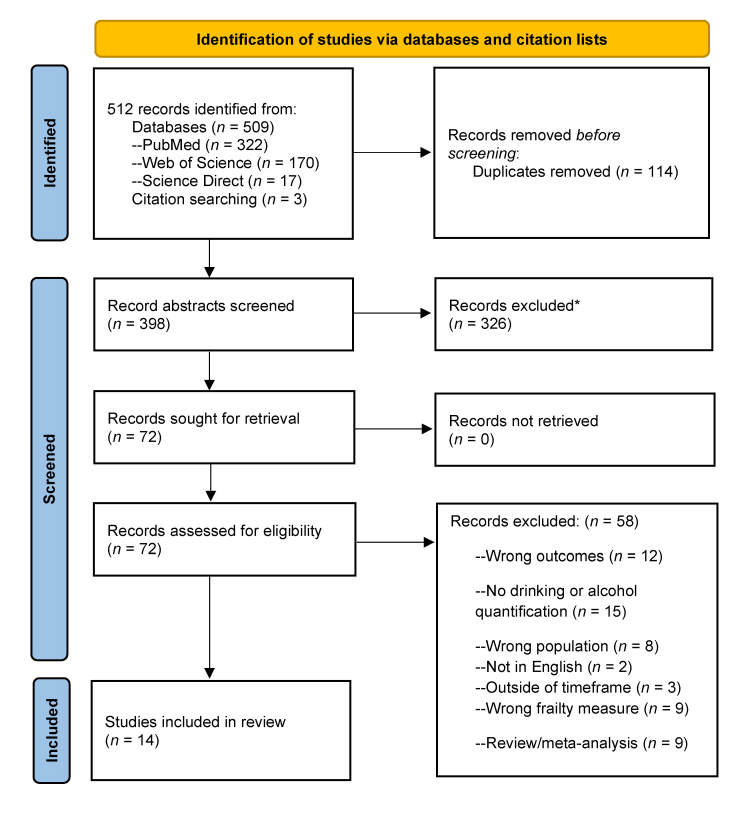
PRISMA 2020 flow diagram for identification of studies of alcohol use and frailty, fractures, and falls among older adults with HIV. *Note:* *Records were excluded at the initial stage of abstract screening due to inappropriate sample composition, exposure, or outcome measures. *Source:* PRISMA flow diagram templates are distributed in accordance with the terms of the Creative Commons Attribution (CC BY 4.0) license. Page MJ, McKenzie JE, Bossuyt PM, et al. The PRISMA 2020 statement: An updated guideline for reporting systematic reviews. *Syst Rev*. 2021;10(1):89. doi:10.1136/bmj.n71

Emerging evidence indicates that comorbid HIV and heavy alcohol use is more detrimental to brain structure and results in higher rates of neurocognitive impairment than either condition alone.^95^–^98^ Binge drinking also poses distinct risks to organ systems^99^ and has been associated with worse global neurocognition, processing speed, delayed recall, and working memory among older PWH.^100^–^102^ Other potential mediators and moderators of the relationship between alcohol use and frailty include ART adherence, polypharmacy, medical and psychiatric comorbidities, and health behaviors, including diet and exercise. The roles of these factors in moderating the effects of alcohol on frailty, fractures, and falls are poorly understood yet are consistent with proposed models (e.g., see Figure 1). Among these, ART adherence is essential to prevent frailty. For example, nonadherence to ART inhibits viral suppression and increases inflammation, which can accelerate the progression of frailty.^103^ Alcohol use has been shown to contribute to ART nonadherence, exacerbating health risks and undermining the effectiveness of HIV treatment.^104^

### Intervention and Prevention Implications

As aging of the PWH population continues, it is important to understand alcohol use patterns, impact on frailty, and common comorbidities, and to consider adaptations to alcohol treatment content and access to services.^105^, ^106^ In the context of AUD treatment, studies of individuals without HIV have found that in contrast to younger adults, older people have more medical comorbidities and worse social support.^107^, ^108^ Providers should take these factors into account to maximize AUD treatment effectiveness for older patients—for example, by enhancing linkages to health and social services. Prior research also has found that older age may be negatively associated with accessing AUD treatment for PWH, including both medications and outpatient specialty care.^109^–^111^ Although frailty is likely a barrier to accessing these services, this issue has not specifically been examined among older PWH.

Alcohol intervention adaptations should consider factors relevant to PWH as well as to older adults in general. For example, motivational interventions for PWH have integrated informational considerations, such as the effect of alcohol on ART adherence, interactions between alcohol and other medications, and alcohol’s impact on common medical and psychiatric comorbidities to which PWH are vulnerable.^112^–^114^ They may also address bothersome health symptoms that PWH might not connect with alcohol use and some of which, such as sleep problems, fatigue, and pain, are especially salient among older PWH.^115^ Stepped-care alcohol treatment models that integrate motivational and contingency management components and can be adapted based on initial treatment response are also promising among older PWH.^116^ More broadly, interventions for older adults that are adapted to take into consideration frailty or disability—for example, through simplification of intervention content, slower pace of treatment, or accommodation of cognitive or sensory deficits—may make alcohol treatment accessible and effective.^117^, ^118^

Apart from interventions to reduce alcohol use, PWH at risk or in the early stages of frailty may benefit from symptom-management or preventive interventions.^119^, ^120^ For example, physical activity or mobility trainings, such as structured cardiovascular or strength-focused exercise, can help preserve functioning.^121^–^123^ Studies have found that exercise-based interventions can reduce falls among older PWoH,^124^, ^125^ and this work is being extended to PWH. For example, Gill et al. conducted a feasibility study of a 10-week online fall prevention intervention tailored for older PWH with alcohol use (mean age 58 years), consisting of weekly virtual group discussions, individual phone check-ins, and home exercises.^126^ The intervention was highly rated by participants, and preliminary analyses suggested that the intervention could reduce the odds of falling as well as alcohol use frequency.

### Limitations of This Review

The effect of alcohol use and related problems on aging and health among PWH is a broad area of research interest. This review focused on a narrow yet important selection of outcomes (i.e., frailty, fractures, and falls), and was not able to address other related topics, such as the relationship of alcohol use to medical comorbidities, polypharmacy, and mortality. This literature search also included only studies from the past 10 years and did not include preclinical (e.g., bone mineral density) studies. Although the review focused on aging PWH, it also included studies with a somewhat broader age range (i.e., studies with the mean ages of participants in their late forties). Finally, the studies varied in their sample size, selection and measurement of exposures and outcomes, and risk of bias. These inherent limitations should be considered when drawing conclusions based on the findings reported here.

## Summary of Conclusions

The population of PWH is rapidly aging, and older PWH are likely to be vulnerable to a range of adverse effects of alcohol use. Overall, the current literature, based on 14 studies that met inclusion criteria, provided evidence that alcohol misuse (i.e., either drinking above recommended limits or AUD) is associated with frailty, fractures, and falls among older PWH. Frailty has historically been conceptualized as physical vulnerability. This review shows that in recent research, frailty has been defined not only by the traditional Fried frailty phenotype (via physical function measures) but also through multidimensional indices and EHR data. This expansion reflects a greater appreciation for the contribution of social, psychosocial, lifestyle, mental health, and other factors that contribute to frailty and vulnerability and which are especially relevant to PWH. Future research that is treatment focused and investigates which interventions may help to reduce alcohol use may help to improve health outcomes for the aging PWH population.

**KEY TAKEAWAYS**

The population of people with HIV (PWH) is rapidly aging and faces unique physical and mental health challenges.

- Alcohol misuse (i.e., either drinking above recommended limits or presence of alcohol use disorder) is prevalent among older PWH and poses substantial risks to health.
- Greater alcohol use is associated with higher risk of frailty, fractures, and falls among older PWH, although the research base remains limited.
- Future research would benefit from longitudinal assessments of the relationship between alcohol use and frailty development, as well as investigation of interventions to reduce risk of frailty, fractures, and falls among older PWH.

## Figures and Tables

**Appendix 1:** Papers Included in the Review of the Associations Between Alcohol Use and Frailty, Fractures, and Falls Among Older People With HIV

First Author	Year	Setting	Sample	Study Design	Alcohol Exposure	Outcomes	Findings	Overall Risk of Bias
Costagliola^71^	2019	Hospital-based open multicenter cohort study; French Hospital Database on HIV	Median age = 49 (IQR 42–58) *N* = 861 67% males	Nested case-control study	Alcohol consumption above 2 units per day; alcohol data recorded at baseline	Fracture diagnosis; fractures prospectively recorded	Alcohol consumption > 2 units/day was higher in PWH with fracture than case-control PWH without fracture (*p* = .002).^a^	High
Crane^64^	2022	CNICS cohort, including PWH in care at 7 sites in the United States	Mean age = 49 (*SD* = 12) *N* = 9,336 85% males	Retrospective cohort study	AUDIT-C in prior year; AUD diagnosis or treatment history in EHR	Modified Fried frailty phenotype (4/5 components)	Being frail was more prevalent among PWH who no longer drank alcohol but had a history of an AUD compared with other categories of alcohol use (*p* < .001).^a^	Low
Justice^19^	2016	VACS of PWH	Mean age = 53 (*SD* = 10.5) *N* = 18,145 100% males	Retrospective cohort study	AUDIT-C score in prior 12 months	VACS Index 1.0	Higher AUDIT-C score was associated with higher VACS Index score (beta = 0.47, 95% *CI* [0.22, 0.73]).	Moderate
Kim^69^	2022	PWH engaged in care in Boston, United States^b^	Mean age = 52 (*SD* = 10) *N* = 251 67% males	Observational cohort study; cross-sectional analysis.	AUDIT-C at baseline; DSM-5 AUD symptoms measured at baseline and follow up	Self-reported fall	Higher AUD symptoms (*OR* = 1.10, 95% *CI* [1.02, 1.18]) or reporting any heavy drinking days (> 6 drinks) was associated with fall risk (*AOR* = 2.24, 95% *CI* [1.21, 4.13]).^c^	Moderate
Kim^68^	2024	PWH engaged in care in Boston, United States	Mean age = 52 (*SD* = 10) *N* = 251 66% males	Observational cohort study; cross-sectional analysis using GEE	Study interviews using TLFB with NIAAA categories for drinking levels (14-day); alcohol use measures were repeated.	Falls, number of falls, fracture, and fracture-related ED visit	Heavy alcohol use was associated with greater odds of fall (*AOR* = 1.49, 95% *CI* [1.08, 2.07]), multiple falls (*AOR* = 1.55, 95% *CI* [1.10, 2.19]), and related ED visit or hospitalization (*AOR* = 1.81, 95% *CI* [1.10, 2.97]).^c^	Moderate
Levitt^61^	2022	NOAH Study; HIV outpatient care setting	Mean age = 48 (*SD* = 10) *N* = 341 71% males	Cross-sectional cohort study	PEth; AUDIT; lifetime alcohol use	DI58; VACS Index 1.0	Recent alcohol use (PEth) moderated the effects of body composition on frailty (DI58), (beta = −6.6 × 10^−3^, *p* = .045).	Moderate
Madkour^60^	2022	NOAH Study; HIV outpatient care setting	Mean age = 48 (*SD* = 10) *N* = 356 69% males	Retrospective cohort study	Lifetime alcohol use trajectories	DI58	Greater increases in alcohol use during midlife were associated with greater frailty (est. beta = 0.183, *SE* = 0.05).	Low
Maffei^37^	2020	NOAH Study; HIV outpatient care setting	Mean age = 48 (*SD* = 10) *N* = 365 71% males	Cross-sectional, prospective study	Lifetime alcohol exposure; recent alcohol exposure (TLFB and PEth)	DI58, Fried frailty phenotype, and VACS 2.0	Lifetime alcohol exposure was positively associated with DI58 (95% *CI* [0.001, 0.006], *p* = .004) and PFI (95% *CI* [0.004, 0.023], *p* = .005).	Low
McMillan^66^	2020	PWH in urban, outpatient HIV clinic	Mean age = 61 (*SD* = 7) *N* = 389 85% males	Cross-sectional, latent-class analysis	Current heavy drinking (> 9 drinks/week for women, > 14 drinks/week for men)	29 clinical indicators extracted from medical records to compute a FI	Current nonheavy alcohol use was associated with lower risk of frailty, compared with abstinence (*OR* = 0.60 (95% *CI* [0.39, 0.94]).	Moderate
Piggott^67^	2017	AIDS Linked to the IntraVenous Experience (ALIVE) community cohort of PWH	Mean age = 48 (*SD* = 7.8) *N* = 415 66% males	Cross-sectional analysis	AUDIT every 6 months	Five original Fried criteria; assessed every 6 months	Hazardous alcohol use was not more prevalent in the prefrail and frail groups (bivariate).^c^	High
Saitz^58^	2018	PWH engaged in care in Boston, United States	Median age = 50 (IQR 44–56) *N* = 250 44% male	Prospective cohort study	TLFB twice per year, with NIAAA cutoffs for heavy alcohol use	Incident fractures in prior year	No associations existed between alcohol consumption and fractures.^c^	Moderate
Womack^59^	2019	VACS	Mean age = 54 (*SD* = 9) *N* = 13,530 96% males	Nested case-control study	AUDIT-C (prior year) closest to fall date	Serious falls (ICD codes and radiology reports)	AUDIT-C score was significantly associated with fall risk (*OR* = 1.30, 95% *CI* [1.23, 1.37]).	Low
Womack^70^	2021	VACS	Mean age = 55 (*SD* = 6) *N* = 26,373 98% males	Cross-sectional cohort analysis	AUD diagnosis	Fragility fracture (defined as hip, vertebral, and upper arm, using ICD) and falls	AUD diagnosis was associated with having a fall (*p* < .001)^a^ and with fragility fracture after a fall (*OR* = 1.49 (95% *CI* [131, 170]).	Low
Womack^40^	2023	VACS	Mean age = 56 (*SD* = 6) *N* = 20,178 98% males	LE-AAF longitudinal cohort analysis	AUD diagnoses and AUDIT-C (prior year)	Osteoporotic and hip fractures	AUD was associated with greater odds of falls (4%; 95% *CI* [4%, 5%]) and fractures (8%; 95% *CI* [7%, 8%]).	Low

a Bivariate association

b This article also included a comparison sample of PWH in St. Petersburg, Russia, results for which are not included in the table due to their younger age (mean age = 39, SD = 6 years).

c Statistical power was potentially limited due to sample size.

*Note*: AOR, adjusted odds ratio; AUD, alcohol use disorder; AUDIT-C, Alcohol Use Disorders Identification Test; CI, confidence interval; CNICS, Centers for AIDS Research Network of Integrated Clinical Systems; DI58, 58-item Deficit Index; DSM, *Diagnostic and Statistical Manual of Mental Disorders*; ED, emergency department; GEE, Generalized Estimating Equations; ICD, International Classification of Diseases; IQR, interquartile range; LE-AAF, Longitudinal Extension of the Average Attributable Fraction; OR, odds ratio; PEth, phosphatidylethanol; PWH, people with HIV; SD, standard deviation; TLFB, timeline follow back; VACS, Veterans Aging Cohort Study.

## References

[1] Marcus JL, Leyden WA, Alexeeff SE (2020). Comparison of overall and comorbidity-free life expectancy between insured adults with and without HIV infection, 2000–2016. JAMA Netw Open.

[2] HIVinfo (2024). HIV and Specific Populations: HIV and Older People.

[3] Williams EC, Hahn JA, Saitz R, Bryant K, Lira MC, Samet JH (2016). Alcohol use and human immunodeficiency virus (HIV) infection: Current knowledge, implications, and future directions. Alcohol Clin Exp Res.

[4] Wing EJ (2016). HIV and aging. Int J Infect Dis.

[5] Desquilbet L, Jacobson LP, Fried LP (2007). HIV-1 infection is associated with an earlier occurrence of a phenotype related to frailty. J Gerontol A Biol Sci Med Sci.

[6] Levett TJ, Cresswell FV, Malik MA, Fisher M, Wright J (2016). Systematic review of prevalence and predictors of frailty in individuals with human immunodeficiency virus. J Am Geriatr Soc.

[7] Molina PE, Simon L, Amedee AM, Welsh DA, Ferguson TF (2018). Impact of alcohol on HIV disease pathogenesis, comorbidities and aging: Integrating preclinical and clinical findings. Alcohol Alcohol.

[8] Molina PE, Gardner JD, Souza-Smith FM, Whitaker AM (2014). Alcohol abuse: Critical pathophysiological processes and contribution to disease burden. Physiology.

[9] Amedee AM, Nichols WA, Robichaux S, Bagby GJ, Nelson S (2014). Chronic alcohol abuse and HIV disease progression: Studies with the non-human primate model. Curr HIV Res.

[10] Satre DD, Sarovar V, Leyden WA (2023). Age group differences in substance use, social support, and physical and mental health concerns among people living with HIV two years after receiving primary care-based alcohol treatment. Aging Ment Health.

[11] Deren S, Cortes T, Dickson VV (2019). Substance use among older people living with HIV: Challenges for health care providers. Front Public Health.

[12] Monroe AK, Lau B, Mugavero MJ (2016). Heavy alcohol use is associated with worse retention in HIV Care. J Acquir Immune Defic Syndr.

[13] Williams EC, McGinnis KA, Edelman EJ (2019). Level of alcohol use associated with HIV care continuum targets in a national U.S. sample of persons living with HIV receiving healthcare. AIDS Behav.

[14] Marshall BDL, Tate JP, McGinnis KA (2017). Long-term alcohol use patterns and HIV disease severity. AIDS.

[15] Edelman EJ, Gordon KS, Glover J, McNicholl IR, Fiellin DA, Justice AC (2013). The next therapeutic challenge in HIV: Polypharmacy. Drugs Aging.

[16] Saitz R (2005). Clinical practice. Unhealthy alcohol use. N Engl J Med.

[17] Park LS, Hernández-Ramírez RU, Silverberg MJ, Crothers K, Dubrow R (2016). Prevalence of non-HIV cancer risk factors in persons living with HIV/AIDS: A meta-analysis. AIDS.

[18] Williams EC, Joo YS, Lipira L, Glass JE (2017). Psychosocial stressors and alcohol use, severity, and treatment receipt across human immunodeficiency virus (HIV) status in a nationally representative sample of U.S. residents. Subst Abus.

[19] Justice AC, McGinnis KA, Tate JP (2016). Risk of mortality and physiologic injury evident with lower alcohol exposure among HIV infected compared with uninfected men. Drug Alcohol Depend.

[20] Dent E, Kowal P, Hoogendijk EO (2016). Frailty measurement in research and clinical practice: A review. Eur J Intern Med.

[21] Rockwood K, Mitnitski A (2007). Frailty in relation to the accumulation of deficits. J Gerontol A Biol Sci Med Sci.

[22] Bortz WM (2002). A conceptual framework of frailty: A review. J Gerontol A Biol Sci Med Sci.

[23] Morley JE, Vellas B, van Kan GA (2013). Frailty consensus: A call to action. J Am Med Dir Assoc.

[24] Althoff KN, Jacobson LP, Cranston RD (2014). Age, comorbidities, and AIDS predict a frailty phenotype in men who have sex with men. J Gerontol A Biol Sci Med Sci.

[25] Retornaz F, Petit N, Darque A (2019). Le phénotype de fragilité chez les personnes vieillissantes avec le VIH: concepts, prévention et enjeux de prise en charge [Frailty phenotype in older people living with HIV: Concepts, prevention and issues]. Geriatr Psychol Neuropsychiatr Vieil.

[26] Bloch M (2018). Frailty in people living with HIV. AIDS Res Ther.

[27] Brothers TD, Kirkland S, Theou O (2017). Predictors of transitions in frailty severity and mortality among people aging with HIV. PLOS One.

[28] Falutz J (2020). Frailty in people living with HIV. Curr HIV AIDS Rep.

[29] Paolillo EW, Saloner R, Montoya JL (2019). Frailty in comorbid HIV and lifetime methamphetamine use disorder: Associations with neurocognitive and everyday functioning. AIDS Res Hum Retrovir.

[30] Ruderman SA, Odden MC, Webel AR (2023). Tobacco smoking and pack-years are associated with frailty among people with HIV. J Acquir Immune Defic Syndr.

[31] Kelly SG, Wu K, Tassiopoulos K, Erlandson KM, Koletar SL, Palella FJ (2019). Frailty is an independent risk factor for mortality, cardiovascular disease, bone disease, and diabetes among aging adults with human immunodeficiency virus. Clin Infect Dis.

[32] Sharma A, Hoover DR, Shi Q (2019). Frailty as a predictor of falls in HIV-infected and uninfected women. Antivir Ther.

[33] Womack JA, Goulet JL, Gibert C (2013). Physiologic frailty and fragility fracture in HIV-infected male veterans. Clin Infect Dis.

[34] Yamada Y, Kobayashi T, Condo A (2022). Prevalence of frailty and prefrailty in people with human immunodeficiency virus aged 50 or older: A systematic review and meta-analysis. Open Forum Infect Dis.

[35] Fried LP, Tangen CM, Walston J (2001). Frailty in older adults: Evidence for a phenotype. J Gerontol A Biol Sci Med Sci.

[36] Mitnitski AB, Mogilner AJ, Rockwood K (2001). Accumulation of deficits as a proxy measure of aging. ScientificWorldJournal.

[37] Maffei VJ, Ferguson TF, Brashear MM (2020). Lifetime alcohol use among persons living with HIV is associated with frailty. AIDS.

[38] McGinnis KA, Justice AC, Moore RD (2022). Discrimination and calibration of the Veterans Aging Cohort Study Index 2.0 for predicting mortality among people with human immunodeficiency virus in north America. Clin Infect Dis.

[39] Akgün KM Tate JP Crothers K An adapted frailty-related phenotype and the VACS index as predictors of hospitalization and mortality in HIV-infected and uninfected individuals J Acquir Immune Defic Syndr 2014 67 4 397 404. doi :10.1097/QAI. 0000000000000341 25202921 10.1097/QAI.0000000000000341 PMC4213242

[40] Womack JA, Murphy TE, Leo-Summers L (2023). Assessing the contributions of modifiable risk factors to serious falls and fragility fractures among older persons living with HIV. J Am Geriatr Soc.

[41] Chang CJ, Chan YL, Pramukti I, Ko NY, Tai TW (2021). People with HIV infection had lower bone mineral density and increased fracture risk: A meta-analysis. Arch Osteoporos.

[42] Gonciulea A, Wang R, Althoff KN (2017). An increased rate of fracture occurs a decade earlier in HIV+ compared with HIV− men. AIDS.

[43] Pramukti I, Lindayani L, Chen YC (2020). Bone fracture among people living with HIV: A systematic review and meta-regression of prevalence, incidence, and risk factors. PLOS One.

[44] Collin F, Duval X, Le Moing V (2009). Ten-year incidence and risk factors of bone fractures in a cohort of treated HIV1-infected adults. AIDS.

[45] Kim TW, Ventura AS, Winter MR (2020). Alcohol and bone turnover markers among people living with HIV and substance use disorder. Alcohol Clin Exp Res.

[46] Mukamal KJ, Mittleman MA, Longstreth WT, Newman AB, Fried LP, Siscovick DS (2004). Self-reported alcohol consumption and falls in older adults: Cross-sectional and longitudinal analyses of the cardiovascular health study. J Am Geriatr Soc.

[47] Sun Y, Zhang B, Yao Q (2022). Association between usual alcohol consumption and risk of falls in middle-aged and older Chinese adults. BMC Geriatr.

[48] Yuan K, Haddad Y, Law R (2023). Emergency department visits for alcohol-associated falls among older adults in the United States, 2011 to 2020. Ann Emerg Med.

[49] Akgün KM, Krishnan S, Tate J (2023). Delirium among people aging with and without HIV: Role of alcohol and Neurocognitively active medications. J Am Geriatr Soc.

[50] Goh SSL, Lai PSM, Tan ATB, Ponnampalavanar S (2018). Reduced bone mineral density in human immunodeficiency virus-infected individuals: A meta-analysis of its prevalence and risk factors. Osteoporos Int.

[51] Jamshaid M, Heidari A, Hassan A (2024). Bone loss and fractures in post-menopausal women living with HIV: A narrative review. Pathogens.

[52] Womack JA, Novick G, Fried T (2018). The beginning of the end: A qualitative study of falls among HIV+ individuals. PLOS One.

[53] Erlandson KM, Plankey MW, Springer G (2016). Fall frequency and associated factors among men and women with or at risk for HIV infection. HIV Med.

[54] Womack JA, Justice AC (2020). The OATH Syndemic: Opioids and other substances, aging, alcohol, tobacco, and HIV. Curr Opin HIV AIDS.

[55] (2024). Covidence systematic review software. Veritas Health.

[56] World Health Organization (2014). Consolidated guidelines on HIV prevention, diagnosis, treatment and care for key populations.

[57] Higgins JPT, Morgan RL, Rooney AA (2024). A tool to assess risk of bias in non-randomized follow-up studies of exposure effects (ROBINS-E). Environ Int.

[58] Saitz R, Mesic A, Ventura AS (2018). Alcohol consumption and bone mineral density in people with HIV and substance use disorder: A prospective cohort study. Alcohol Clin Exp Res.

[59] Womack JA, Murphy TE, Rentsch CT (2019). Polypharmacy, hazardous alcohol and illicit substance use, and serious falls among PLWH and uninfected comparators. J Acquir Immune Defic Syndr.

[60] Madkour AS, Felker-Kantor E, Welsh DA, Molina PE, Theall KP, Ferguson T (2022). Lifetime alcohol use trajectories and health status among persons living with HIV (PLWH). J Stud Alcohol Drugs.

[61] Levitt DE, Simon L, Lin HY (2022). Alcohol use, physical activity, and muscle strength moderate the relationship between body composition and frailty risk among people living with HIV. Alcohol Clin Exp Res.

[62] Skinner HA, Sheu WJ (1982). Reliability of alcohol use indices. The lifetime drinking history and the MAST. J Stud Alcohol.

[63] Sobell LC, Sobell MB, Litten RZ, Allen JP (1992). Timeline follow-back: A technique for assessing self-reported alcohol consumption. Timeline Follow-Back: A Technique for Assessing Self-Reported Alcohol Consumption.

[64] Crane HM, Ruderman SA, Whitney BM (2022). Associations between drug and alcohol use, smoking, and frailty among people with HIV across the United States in the current era of antiretroviral treatment. Drug Alcohol Depend.

[65] Sung M, Gordon K, Edelman EJ, Akgün KM, Oursler KK, Justice AC (2021). Polypharmacy and frailty among persons with HIV. AIDS Care.

[66] McMillan JM, Gill MJ, Power C, Fujiwara E, Hogan DB, Rubin LH (2020). Comorbidities in older persons with controlled HIV infection: Correlations with frailty index subtypes. AIDS Patient Care STDs.

[67] Piggott DA, Muzaale AD, Varadhan R (2017). Frailty and cause-specific hospitalization among persons aging with HIV infection and injection drug use. J Gerontol A Biol Sci Med Sci.

[68] Kim TW, Bertholet N, Magane KM (2024). Alcohol consumption and illicit drug use: Associations with fall, fracture, and acute health care utilization among people with HIV infection. J Acquir Immune Defic Syndr.

[69] Kim TW, Heeren TC, Samet JH (2022). Alcohol and falls among people with HIV infection: A view from Russia and the United States. Alcohol Clin Exp Res.

[70] Womack JA, Murphy TE, Ramsey C (2021). Brief report: Are serious falls associated with subsequent fragility fractures among veterans living with HIV?. J Acquir Immune Defic Syndr.

[71] Costagliola D, Potard V, Lang S (2019). Impact of antiretroviral drugs on fracture risk in HIV-infected individuals: A case-control study nested within the French Hospital Database on HIV (FHDH-ANRS CO4). J Acquir Immune Defic Syndr.

[72] Agabio R, Campesi I, Pisanu C, Gessa GL, Franconi F (2016). Sex differences in substance use disorders: Focus on side effects. Addict Biol.

[73] Erol A, Karpyak VM (2015). Sex and gender-related differences in alcohol use and its consequences: Contemporary knowledge and future research considerations. Drug Alcohol Depend.

[74] Blanco JR, Barrio I, Ramalle-Gómara E (2019). Gender differences for frailty in HIV-infected patients on stable antiretroviral therapy and with an undetectable viral load. PLOS One.

[75] Gustafson DR, Shi Q, Thurn M (2024). Frailty-related factors among women living with and without HIV aged 40 years and older. The Women’s Interagency HIV Study. J Frailty Aging.

[76] Psomas CK, Hoover DR, Shi Q (2022). Polypharmacy is associated with falls in women with and without HIV. J Acquir Immune Defic Syndr.

[77] Sharma A, Hoover DR, Shi Q (2021). High frequency of recurrent falls among prefrail and frail women with and without HIV. J Acquir Immune Defic Syndr.

[78] Dela Cruz JJ, Karpiak SE, Brennan-Ing M (2014). Health outcomes for older Hispanics with HIV in New York City using the Oaxaca Decomposition Approach. Glob J Health Sci.

[79] Jimenez DE, Weinstein ER, Batsis JA (2022). “Me dieron vida”: The effects of a pilot health promotion intervention to reduce cardiometabolic risk and improve behavioral health among older Latinos with HIV. Int J Environ Res Public Health.

[80] Rodríguez-Mañas L, Féart C, Mann G (2013). Searching for an operational definition of frailty: A Delphi method based consensus statement: The frailty operative definition-consensus conference project. J Gerontol A Biol Sci Med Sci.

[81] Walston J, Hadley EC, Ferrucci L (2006). Research agenda for frailty in older adults: Toward a better understanding of physiology and etiology: Summary from the American Geriatrics Society/National Institute on Aging Research Conference on Frailty in Older Adults. J Am Geriatr Soc.

[82] Greene M, Justice AC, Covinsky KE (2017). Assessment of geriatric syndromes and physical function in people living with HIV. Virulence.

[83] Perilli M, Toselli F, Franceschetto L (2023). Phosphatidylethanol (PEth) in blood as a marker of unhealthy alcohol use: A systematic review with novel molecular insights. Int J Mol Sci.

[84] Kojima G, Liljas A, Iliffe S, Jivraj S, Walters K (2018). A systematic review and meta-analysis of prospective associations between alcohol consumption and incident frailty. Age Ageing.

[85] Ruderman SA, Drumright LN, Delaney JAC (2024). Evaluating the sick quitting hypothesis for frailty status and reducing alcohol use among people with HIV in a longitudinal clinical cohort study. J Assoc Nurses AIDS Care.

[86] Bobrow K, Xia F, Hoang T, Valcour V, Yaffe K (2020). HIV and risk of dementia in older veterans. AIDS.

[87] Lam JO, Lee C, Gilsanz P (2022). Comparison of dementia incidence and prevalence between individuals with and without HIV infection in primary care from 2000 to 2016. AIDS.

[88] Liu Z Han L Gahbauer EA Allore HG Gill TM Joint trajectories of cognition and frailty and associated burden of patient-reported outcomes J Am Med Dir Assoc 2018 19 4 304 309.e2 10.1016/j.jamda.2017.10.010 29146224 PMC6054444

[89] Rieker JA, Reales JM, Muiños M, Ballesteros S (2022). The effects of combined cognitive-physical interventions on cognitive functioning in healthy older adults: A systematic review and multilevel meta-analysis. Front Hum Neurosci.

[90] Sullivan EV, Zahr NM, Zhao Q, Pohl KM, Sassoon SA, Pfefferbaum A (2024). Contributions of cerebral white matter hyperintensities to postural instability in aging with and without alcohol use disorder. Biol Psychiatry Cogn Neurosci Neuroimaging.

[91] Dudley MT, Borkum M, Basera W, Wearne N, Heckmann JM (2019). Peripheral neuropathy in HIV patients on antiretroviral therapy: Does it impact function?. J Neurol Sci.

[92] de Almeida LL, Ilha TASH, de Carvalho JAM (2020). Sarcopenia and its association with vertebral fractures in people living with HIV. Calcif Tissue Int.

[93] Chatha H, Sammy I, Hickey M, Sattout A, Hollingsworth J (2018). Falling down a flight of stairs: The impact of age and intoxication on injury pattern and severity. Trauma.

[94] Taylor B, Irving HM, Kanteres F (2010). The more you drink, the harder you fall: A systematic review and meta-analysis of how acute alcohol consumption and injury or collision risk increase together. Drug Alcohol Depend.

[95] Pfefferbaum A, Zhao Q, Pohl KM, Sassoon SA, Zahr NM, Sullivan EV (2024). Age-accelerated increase of white matter hyperintensity volumes is exacerbated by heavy alcohol use in people living with HIV. Biol Psychiatry.

[96] Rosenbloom MJ, Sullivan EV, Pfefferbaum A (2010). Focus on the brain: HIV infection and alcoholism: Comorbidity effects on brain structure and function. Alcohol Res Health.

[97] Rothlind JC, Greenfield TM, Bruce AV (2005). Heavy alcohol consumption in individuals with HIV infection: Effects on neuropsychological performance. J Int Neuropsychol Soc.

[98] Sullivan EV, Pfefferbaum A (2019). Brain-behavior relations and effects of aging and common comorbidities in alcohol use disorder: A review. Neuropsychology.

[99] Molina PE, Nelson S (2018). Binge drinking’s effects on the body. Alcohol Res.

[100] Paolillo EW, Saloner R, Kohli M (2022). Binge drinking relates to worse neurocognitive functioning among adults aging with HIV. J Int Neuropsychol Soc.

[101] Testino G, Fagoonee S, Caputo F, Pellicano R (2021). The early identification of alcohol use disorders and liver injury: Proposal for a diagnostic algorithm. Panminerva Med.

[102] Britton MK, Porges EC, Bryant V, Cohen RA (2021). Neuroimaging and cognitive evidence for combined HIV-alcohol effects on the central nervous system: A review. Alcohol Clin Exp Res.

[103] Kalichman SC, Grebler T, Amaral CM (2013). Intentional non-adherence to medications among HIV positive alcohol drinkers: Prospective study of interactive toxicity beliefs. J Gen Intern Med.

[104] Musinguzi N, Castillo-Mancilla J, Morrow M (2019). Antiretroviral therapy adherence interruptions are associated with systemic inflammation among Ugandans who achieved viral suppression. J Acquir Immune Defic Syndr.

[105] Althoff KN, Smit M, Reiss P, Justice AC (2016). HIV and ageing: Improving quantity and quality of life. Curr Opin HIV AIDS.

[106] Mannes ZL, Bryant VE, Burrell LE (2019). The prevalence and patterns of substance use by birth cohort among HIV-positive adults in Florida. Aging Ment Health.

[107] Al-Otaiba Z, Epstein EE, McCrady B, Cook S (2012). Age-based differences in treatment outcome among alcohol-dependent women. Psychol Addict Behav.

[108] Satre DD, Chi FW, Mertens JR, Weisner CM (2012). Effects of age and life transitions on alcohol and drug treatment outcome over nine years. J Stud Alcohol Drugs.

[109] Oldfield BJ, McGinnis KA, Edelman EJ (2020). Predictors of initiation of and retention on medications for alcohol use disorder among people living with and without HIV. J Subst Abuse Treat.

[110] Satre DD, DeLorenze GN, Quesenberry CP, Tsai A, Weisner C (2013). Factors associated with treatment initiation for psychiatric and substance use disorders among persons with HIV. Psychiatr Serv.

[111] Davy-Mendez T, Sarovar V, Levine-Hall T (2021). Treatment for alcohol use disorder among persons with and without HIV in a clinical care setting in the United States. Drug Alcohol Depend.

[112] Hasin DS, Aharonovich E, Zingman BS (2022). HealthCall: A randomized trial assessing a smartphone enhancement of brief interventions to reduce heavy drinking in HIV care. J Subst Abuse Treat.

[113] Madhombiro M, Kidd M, Dube B (2020). Effectiveness of a psychological intervention delivered by general nurses for alcohol use disorders in people living with HIV in Zimbabwe: A cluster randomized controlled trial. J Int AIDS Soc.

[114] Parry CDH, Myers B, Londani M (2023). Motivational interviewing and problem-solving therapy intervention for patients on antiretroviral therapy for HIV in Tshwane, South Africa: A randomized controlled trial to assess the impact on alcohol consumption. Addiction.

[115] Bahji A, Gordon KS, Crystal S (2023). Factors associated with bothersome symptoms in individuals with and without HIV who report alcohol use. AIDS Behav.

[116] Edelman EJ, Dziura J, Deng Y (2025). Integrated stepped alcohol treatment with contingency management for unhealthy alcohol use among people with HIV: A randomized controlled trial. J Acquir Immune Defic Syndr.

[117] Satre DD (2015). Alcohol and drug use problems among older adults. Clin Psychol Sci Pract.

[118] Satre DD, McCrady BS, Epstein EE (2013). Treatment of older adults. Addiction, A Comprehensive Guidebook.

[119] Erlandson KM, Piggott DA (2021). Frailty and HIV: Moving from characterization to intervention. Curr HIV AIDS Rep.

[120] Kehler DS, Milic J, Guaraldi G, Fulop T, Falutz J (2022). Frailty in older people living with HIV: Current status and clinical management. BMC Geriatr.

[121] Billot M, Calvani R, Urtamo A (2020). Preserving mobility in older adults with physical frailty and sarcopenia: Opportunities, challenges, and recommendations for physical activity interventions. Clin Interv Aging.

[122] Erlandson KM, MaWhinney S, Wilson M (2018). Physical function improvements with moderate or high-intensity exercise among older adults with or without HIV infection. AIDS.

[123] Lopez P, Pinto RS, Radaelli R (2018). Benefits of resistance training in physically frail elderly: A systematic review. Aging Clin Exp Res.

[124] Gillespie LD, Robertson MC, Gillespie WJ (2012). Interventions for preventing falls in older people living in the community. Cochrane Database Syst Rev.

[125] Sun M, Min L, Xu N, Huang L, Li X (2021). The effect of exercise intervention on reducing the fall risk in older adults: A meta-analysis of randomized controlled trials. Int J Environ Res Public Health.

[126] Gill SV, Shin D, Kim TW (2025). A fall prevention feasibility trial for people with HIV and alcohol use. OTJR (Thorofare NJ).

